# Epidemiology of Herpes Zoster Infection among Patients Treated in Primary Care Centres in the Valencian Community (Spain)

**DOI:** 10.1186/1471-2296-11-33

**Published:** 2010-05-06

**Authors:** Ana M Cebrián-Cuenca, Javier Díez-Domingo, María San-Martín Rodríguez, Joan Puig-Barberá, Jorge Navarro-Pérez

**Affiliations:** 1 Centro de Salud de Ayora, Argentina Avenue, Ayora, Valencia, Spain; 2 Centro Superior de Investigaciones en Salud Pública de Valencia (CSISP), Av. Cataluña 21. 46020, Valencia, Spain; 3 Departamento Médico Sanofi Pasteur MSD, Paseo de la Castellana, 141, Madrid, Spain; 4 Centro de Salud Pública de Castellón, Avda. del Mar, 12, 12003 Castellón de la Plana, Valencia, Spain; 5 Dirección Médica de Atención Primaria del Departamento 5. Hospital Clínico Universitario de Valencia, Blasco Ibáñez Avenue, 46020, Valencia, Spain

## Abstract

**Background:**

There is little available data regarding the epidemiology of herpes zoster (HZ) in Spain. This study's main goal was to estimate the annual incidence of HZ in the Autonomous Community of Valencia.

**Methods:**

From December 1^st ^2006 to December 1^st ^2007, a prospective study was carried out in 24 primary health care centres that together provide care for a population of 36,030 persons aged >14 years. We included all adult patients with a clinical diagnosis of HZ who were seen at these centres during the one-year study period. Demographic (i.e., age, gender, and area of residence) and clinical data were also collected from these patients.

**Results:**

A total of 146 cases of HZ were identified during the study period. The annual incidence of HZ was 4.1/1,000 individuals >14 years of age (95% confidence interval [CI]: 3.4-4.7). Cases of HZ were predominantly unilateral and most commonly affected women and people living in rural areas. The most frequently reported symptoms were pain, dysesthesia and itching. A total of 46% of patients also had underlying illnesses (e.g., chronic diseases and/or malignancy) and 24% of patients experienced complications, which were mostly ocular in nature. A total of 91% of patients were treated with antiviral drugs. The median time from symptoms onset to diagnosis was 6.3 days (range: 2.0-8.3).

**Conclusions:**

HZ is a common illness in our region (especially in the older population) that causes a significant clinical burden on primary care providers.

## Background

Herpes zoster (HZ) is caused by the reactivation of latent infection with varicella zoster virus (VZV) after primary varicella infection. The condition is characterised by the localised eruption of vesicular lesions following the trajectory of a sensory nerve as well as pain and inflammation of the affected nerve root [1].

The most frequent and debilitating complication of HZ is postherpetic neuralgia (PHN), which is a form of neuropathic pain that appears in the dermatomes affected by the VZV infection. The pain associated with PHN is intense and disabling, and PHN has a significant impact upon patients' quality of life [2].

The incidence and severity of both HZ and PHN increase significantly with age [3-7]. The annual incidence of HZ that have been reported in population-based studies that have been published in several countries ranges from 1.2 to 4.8 cases per 1000 inhabitants/year. The lifetime risk of zoster is estimated to be 10-30% and increases markedly with age, occurring in up to 50% in people who live to be 85 years of age [3,6-11]. In turn, PHN affects 10-70% of patients with HZ. This wide range is partly due to differences in the definitions of PHN that are used in various studies and to the ages of the study populations from which these estimates were derived. In older patients, the prevalence of PHN among patients with HZ is likely closer to the upper end of that range [2].

An increase in the incidence of HZ has been observed in some countries through epidemiological surveillance systems. This increase could be explained by the increase in the use of immunosuppressive agents and the development and clinical use of biological agents with immunosuppressive capacity as well as by other factors that are still not well known [12,13]. The aging of the population in the industrialised world [12,13] might have a limited role in this phenomenon since most of the studies in this setting present age-adjusted rates. Based on a mathematical model, it has also been speculated that the prevalence of HZ may increase in populations that have a high rate of paediatric vaccination against VZV [5], but thus far, this theoretical model has not been confirmed by epidemiological data. Moreover, some very recent publications have concluded that the impact that VZV vaccination programs have on the incidence of HZ is uncertain [14,15].

There is little information available concerning the epidemiology of HZ in Spain, and studies are needed to evaluate the epidemiological and pharmacoeconomical impact of this disease. The purpose of the present study was to estimate the incidence as well as the clinical and epidemiological characteristics of HZ in Spain.

## Methods

During a 1-year period (from December 1^st^, 2006 to December 1^st^, 2007), a prospective study was carried out in 24 primary care GP offices that are part of the public healthcare system. We randomly selected the offices from rural, urban, and semiurban areas in the province of Valencia that we felt were representative of the Autonomous Community of Valencia (Spain).

### Sample size calculation

Based on an expected incidence of HZ in the adult population of 0.4% and a precision of 0.08%, we calculated that a minimum population of 23,600 people >14 years of age should be included in the population that was monitored [6]. The study population assigned to the participating investigators included 36,030 individuals. This population was comprised of 21,500 patients who were 15-49 years of age, 4,893 patients who were 50-59 years of age, 4,057 patients who were 60-69 years of age of age, and 5,580 patients who were ≥ 70 years of age. Additionally, 51% of this population was female. The age and genders of the patients that were recruited to participate in our study were similar to those that are observed in general population (based on data from national registries) [16].

### Study population

During the study period, all patients who were >14 years of age and had a clinical diagnosis of HZ were considered to be eligible for inclusion. All study participants signed an informed consent form prior to inclusion in the study.

For each patient who agreed to participate and signed the consent form, a case report form was completed that included questions regarding patients' demographic data, information relating to personal history and previous clinical disorders that are thought to increase the risk of HZ and/or its complications, and the clinical characteristics of the HZ episode and its associated complications. This information was obtained by an interview with the patient and review of his/her medical history. Patients with HZ who declined to participate in the study were anonymously counted as HZ cases to allow a more accurate estimation of the incidence of the disease.

The intensity of the pain that the patients experienced and the degree to which it interfered with their activities of daily living were evaluated using a validated and previously published quantitative scale called the Zoster Brief Pain Inventory (BPI) [17]. The BPI consists of nine questions that are divided into two parts: the first part (which is scored from 0 to 10) assesses the intensity of pain experienced by the patient during the preceding 24 hours, the intensity of pain experienced by the patient at the time of questionnaire administration, the anatomical location of the referred pain, the use of analgesics to combat the pain, and the degree to which those analgesics relieved their pain. The second part of the inventory (which is likewise scored from 0 to 10) addresses the degree to which patients' pain interferes with their general activity level, mood state, mobility, ability to perform their job, relationships with other people, sleep, and quality of life. With the purpose of this study we considered as "ocular complications" all nerve ophthalmic localizations.

The study was approved by the Clinical Research Ethics Committee of the Dirección General de Salud Pública/Centro Superior de Investigación en Salud Pública (CSISP) de la Comunidad Valenciana.

### Statistical analysis

The annual incidence of HZ was calculated from the episodes that were recorded during the study period. The appearance of new lesions at the same location was considered to be a relapse of the same episode and was not counted as a new case. The total number of individuals assigned to the quota of the participating investigators at the start of the study was used as the denominator. There was no substantial variation in the number of individuals assigned to the participating investigators during the study period [there was relative increase equal to 0.6% (216 persons) from study initiation to end of follow-up]. The incidence was calculated globally by gender and by the predefined age groups (<50 years, 50-59 years, 60-69 years, and ≥ 70 years of age). Comparisons between groups were performed using Student's t-test for continuous variables with a normal distribution and the Mann-Whitney U-test for non-normally distributed continuous variables. The chi-squared test or Fisher's exact test were used to compare the distribution of discrete variables between groups. All p-values < 0.05 were considered to be statistically significant. All statistical analyses were performed using the SPSS software package, version 12.0 (SPSS Inc., Chicago, IL, USA).

## Results

A total of 146 cases of HZ were recorded during the study period. Of these 146 patients, 130 (89%) consented to participate in the study. The patients that we included in our study were significantly younger than those who refused to be recruited (61 vs. 73 years, p < 0.01), but the remaining baseline characteristics were not significantly different in both groups. Of the patients included in our study, 64% were women and 38.5% lived in a rural setting. The mean age of included patients was 61.1 years (SD: 17.7). Additionally, 46% of patients presented with 1 or more background clinical conditions, which, because of its physiopathology or suggested treatment, was considered to predispose patients to the development of HZ or its complications, specifically PHN. The most frequent underlying condition with which our patients presented was chronic disease (30% of the patients), which included diabetes mellitus, chronic obstructive pulmonary disease, moderate or severe asthma, rheumatoid arthritis, chronic liver disease, chronic kidney disease, congenital heart disease, and systemic lupus erythematosus. The second most common predisposing condition with which our patients presented was active malignancy (9%). Prodromic pain was reported by 41% of patients. A total of 33% of these patients described their pain as severe and 49% described it as moderate. Table 1 lists the baseline characteristics of the included patients.

**Table 1:** Baseline characteristics of the included patients.

Variable	n = 130
Age in years, mean (SD)	61.1 (17.7)
Female gender, n (%)	83 (63.8)
**Area of residence**	
Urban, n (%)	41 (31.5)
Semiurban, n (%)	39 (30)
Rural, n (%)	50 (38.5)
**Predisposing clinical conditions†**	60 (46.2)
Immunosuppressant◇ use, n (%)	3 (2.3)
Malignancy, n (%)	12 (9.2)
HIV, n (%)	0
Transplant, n (%)	1 (0.8)
Chronic disease*, n (%)	39 (30)
Trauma/burns/radiotherapy, n (%)	3 (2.3)
Other**	6 (4.6)
Not reflected in clinical history, n (%)	2 (1.5)
**Complications†, n (%)**	31 (23.8)
Ocular, n (%)	11 (8.5)
Bacterial superinfection, n (%)	7 (5.4)
Dysgeusia, n (%)	4 (3.1)
Hypoacusis, vertigo, tinnitus, n (%)	2 (1.5)
Dissemination, n (%)	2 (1.5)
Involvement of other organs, n (%)	1 (0.8)
Other‡, n (%)	12 (9.2)
**Use of antiviral agents, n (%)**	119 (91.5)
**Time elapsed from symptom onset to diagnosis, days (SD)**	6.3 (5.8)

SD: Standard deviation.

HIV: Human immunodeficiency virus.

◇We considered the administration of oral or systemic corticosteroids or chemotherapy treatment to constitute immunosuppressant use.

† Each patient may have more than one baseline clinical condition/complication.

* Chronic disease was defined as diabetes mellitus, chronic obstructive pulmonary disease, moderate or severe asthma, rheumatoid arthritis, chronic liver disease, chronic kidney disease, congenital heart disease, and systemic lupus erythematosus.

** Other risk factors refer to multisystem atrophy, major depression, postsurgical stress, influenza, and polysubstance abuse.

‡ Other complications refer to lymphadenopathy, activity limitation, fungal infection in the HZ area, allergic reactions to treatment, weight loss, anorexia, vomiting, and diarrhoea.

The most frequent location of HZ lesions was the thoracic region (42.3%) and most patients (96%) had a unilateral presentation of their symptoms. A total of 78% of patients reported experiencing neuropathic-type pain in the dermatome in which the skin lesions developed. The mean pain intensity that the patients experienced, as assessed by the BPI, was scored as a 3.2 (SD: 2.3). The mean degree to which the pain interfered with patients' activities of daily living, which was also assessed by the BPI, was scored as a 3.2 (SD: 2.7). Dysesthesia was present in 87% of patients. Table 2 shows the characteristics of the initial HZ episode that was experienced by the study patients. A total of 24% of patients experienced complications, which most often presented as ocular problems (mainly conjunctivitis and blepharitis).

**Table 2:** Characteristics of the initial herpes zoster episode.

	n = 130
**Location**	
Ophthalmic, n (%)	13 (10)
Cervical, n (%)	16(12.3)
Thoracic, n (%)	55 (42.3)
Lumbar, n (%)	27 (20.7)
Sacral, n (%)	19 (14.6)
Unilateral, n (%)	125 (96.2)
Bilateral, n (%)	5 (3.8)
**Other symptoms**	
Prodromic pain, n (%)	53 (40.8)
Pain at presentation, n (%)	101 (77.7)
Dysesthesia, n (%)	113 (86.9)
Pruritus, n (%)	101 (77.7)
Malaise, n (%)	70 (53.8)
Headache, n (%)	46 (35.4)
Fever, n (%)	16 (12.3)
Other^#^, n (%)	7 (5.4)

^# ^Allodynia, diarrhoea, bone pain, irritability, paresthesias, and vomiting.

In 91.5% of patients, antiviral drugs were prescribed to treat the HZ episode. The prescription of antiviral drugs was not associated with the presence of pain at the time of diagnosis or the time that had elapsed since the appearance of symptoms (p > 0.05). The median time that elapsed between the appearance of the viral exanthem to diagnosis was 4.0 days (IQR: 2.0-8.3).

The estimated global annual incidence of HZ in this observational cohort study was 4.1 per 1000 individuals (95% confidence interval [CI]: 3.4-4.7). By age group, the annual incidence of HZ, was significantly higher in patients 50-59 years of age (6.7/1,000 inhabitants; 95%: CI 4.4-9) and in those ≥ 70 years of age (11.1/1,000 inhabitants; 95% CI: 8.3-13.9), compared to those patients aged from 60 to 69 years (annual incidence of 5.2/1000 inhabitants [95% CI: 3-7.4] and < 50 years (1.3/1,000 inhabitants [95% CI: 0.9-1.8]). The annual incidence was also higher in females (4.5/1,000 inhabitants; 95% CI: 3.5-5.4) than it was in males (2.7/1,000 inhabitants; 95% CI: 1.9-3.5; p = 0.005). Significant differences were also observed in the incidence of pain by age group, which varied from 1/1,000 inhabitants for individuals <50 years of age to 6.7/1,000 inhabitants for individuals ≥ 70 years. The percentage of patients experiencing pain at the time of diagnosis, by age group, was 75.9% in the subjects who were <50 years of age, 80% in the patients who were 50-59 years of age, 90% in the patients who were 60-69 years of age, and 72.5% in the patients who were ≥ 70 years of age.

The annual incidence of HZ also differed significantly (p < 0.001) by place of residence. The annual incidence was greater in the rural setting (8.8/1,000 for individuals >14 years of age; 95% CI: 6.4-11.2) than it was in the semiurban (3.1/1,000 individuals >14 years of age; 95% CI: 2.2-4) or urban setting (3.1/1,000 individuals >14 years; 95% CI: 2.2-3.9).

## Discussion

The estimated annual incidence of HZ in our study was 4.1 per 1,000 persons >14 years of age. Other studies that have been carried out in our country in the primary care setting have reported similar results. In a study performed in Navarra, which involved a retrospective review of the computer-based primary care patient records of patients who were seen between 2005 and 2006 [18], the mean annual incidence was 4.25 per 1,000 inhabitants (0- ≥ 75 years). In the Autonomous Community of Madrid, an average annual incidence of HZ was 2.49-3.59 per 1,000 persons (0- ≥ 85 years) was estimated based on reporting by the local Sentinel Physicians Network between 1997 and 2004 [19]. Unlike the aforementioned studies, the present study was specifically designed to *prospectively *estimate the incidence of HZ in real-life practices.

In a recent retrospective population-based study carried out in the United States in which new cases of HZ that were identified by medical records review, the observed annual incidence of HZ was 3.6/1,000 adults >22 years of age [13]. Another study from the US, in which administrative data were used as the data source, reported an incidence of 3.2 per 1,000 inhabitants in the general population (0- ≥ 80 years) [20]. In a recent study carried out in the United States conducted by Oxman and Cols [4] in people ≥ 60 years was observed an HZ incidence of 11.12/1,000 persons/year in the control arm of the clinical trial. By age group, Oxman and Cols reported a HZ incidence of 10.8/1,000 persons/year among individuals 60-69 years of age and 11.5/1,000 persons/year among individuals ≥ 70 years of age in the control arm of the clinical trial. While the incidence of HZ in the group ≥ 70 is similar to our data (11.1/1,000 inhabitants), the HZ incidence in the group 60-69 years is higher in Oxman's study than our study (5.2/1,000 persons). The reason for this discrepancy is not well understood, but it could be influenced by the different designs of these studies (i.e., experimental [4] versus observational [13,20]). The study by Oxman and Cols' [4] group included more patients (n = 19,276 patients were included in the control arm) than the other two studies combined [13,20] (n = 10,821) and moreover, the inclusion/exclusion criteria of the trial were not very restrictive (exclusion was limited to immunosuppressed individuals). The differing objectives of the studies (evaluation of the effectiveness of an intervention [3] vs. an estimation of a population-based incidence [13,20]) may also have contributed to the differences in the findings of these studies.

Results from studies that have been carried out in European countries are also consistent with those obtained in our study. An incidence of 5.23/1,000 persons/year among individuals ≥ 50 years of age was reported in a retrospective study that was undertaken in the United Kingdom between 2000 and 2006 [21]. An incidence of 4.8/1,000 persons/year (0->74 years) was observed in a prospective French registry [22] that was specifically designed to analyse the epidemiology of HZ in that country.

It should be highlighted that the results of descriptive epidemiological studies are highly dependent on the methodology that is used. Accordingly, studies that use the voluntary reporting of cases and/or review of previous databases as their sources of information have limitations that are fundamentally related to the coding of diagnoses, and these limitations can influence the study findings. Moreover, the results of studies in which recruitment is carried out in medical outpatient clinics may be influenced by the characteristics of the healthcare system of the country in which the study is performed. For example, prospective studies that have been performed in Holland (3.2 cases/1,000 persons {0->75 years}) [23] and the United Kingdom (0.6-4.3/1,000 persons-year {all ages}) [24] have reported lower incidences of HZ compared to our estimated incidence. In the latter study, the lower incidence that was observed may have been due to a low rate of inclusion of HZ patients, the exclusion of patients whose rashes were too old to be confirmed by laboratory tests (i.e., more than 7 days after onset), and the inclusion of a cohort of patients whose ethnic diversity was not representative of that of the general population. This might have biased the HZ incidence that was observed because it has been suggested by other studies that non-Caucasian ethnic groups may have a lower risk of HZ than Caucasians [6].

The characteristics of our patients and the clinical presentation of their HZ episodes are consistent with those that have been reported in other large-scale studies that have been carried out in developed countries [21-27]. Similar to our study, in studies that have been performed in Spain [18] and the United States [13,20], over half of the included patients were >60 years of age, and over two-thirds were >50 years of age. Moreover, in most studies [13,18-23,28], the HZ incidence was higher in females than it was in males. The reasons for these gender-related differences are not well understood. In our study, the age distribution was similar among males and females, so an age bias does not explain the gender-related differences in HZ incidence that we observed. Some authors have suggested that differences in the utilisation of health care resources between males and females or different immune responses to the latent viral infection may offer a partial explanation for these findings [28]. The observation of a higher HZ incidence in women is controversial because an association between repeated exposure to VZV and a reduction in the risk of developing HZ [5,28] has been proposed and is currently under debate. Women typically have more contact with children (including children with VZV) than men do [29,30]. Thus, if there is an association between repeated exposure to VZV and a reduction in the risk of developing HZ, one would expect that women would have a lower incidence of HZ than men [1].

There were not any significant differences between the age and sex distribution of patients in the rural, semiurban, and urban settings. Therefore, the higher incidence of HZ that was observed in the rural areas could be partially attributable in part to a higher consultation rate among the rural population due to the lower number of patients that are assigned to reference GPs in rural practices.

In our study, we found that people ≥ 70 years of age reported pain at the time of diagnosis less frequently than the other age groups. Other authors have described that with advancing age, the nociceptive pathway undergoes degenerative changes, which mainly consist of axonal loss. This age-related nociceptive pathway degeneration probably explains why elderly patients tend to under-report pain in many medical conditions, including myocardial infarction, fractures, and arthritis and also probably plays a key role in the development of neuropathic pain in patients with HZ [31]. As in other studies [13,22], we found that the most frequent location of HZ lesions was the thoracic region (42.3%), followed by the head and neck (22.3%).

Almost half (46%) of the patients in our study had some type of underlying illness, but only 11.5% exhibited immunosuppression related to malignancies or immunosuppressive therapy. It can be hypothesised that the prevention of HZ and/or its complications in a population with a high prevalence of chronic medical conditions might allow patients to avoid potential interference with the treatment of their chronic diseases or a worsening of those chronic diseases. These results, as well as those published by Oxman [4] (in a cohort of non-immunocompromised individuals) support the idea that there is an important subgroup of patients, particularly the elderly patients that could be potential candidates for HZ prevention through vaccination [13].

Non-pain-related complications occurred in 23.8% of the patients. Although this percentage is higher than the rates that have been observed in previous studies [13], the distribution of the complications we observed was similar to the distribution of symptoms that has been described in other studies, with ocular complications being the most frequently reported type of complication. The higher proportion of complications that were observed in this study as compared to the study published by Yawn et al. may be explained, in part, by the different percentages of immunocompromised patients that were included in the two studies (12% vs. 8%, respectively) (13).

The effectiveness of antiviral agents in treating HZ is a subject of controversy because there is no consensus regarding their potential capacity to reduce the occurrence of severe complications, specifically postherpetic neuralgia [21,32]. In a recent review to investigate the effectiveness of antiviral agents in preventing PHN, the authors concluded that oral acyclovir did not significantly reduce the incidence of PHN and that there was insufficient evidence from randomised controlled trials to determine whether other antiviral treatments prevent PHN [33]. When such drugs are used, it has been recommended that they should be administered as early as possible in the course of the illness in order to be effective [34]. In our study, antivirals were prescribed to 91.5% of the patients, and the median time that elapsed from the appearance of the viral exanthem to the initiation of antiviral therapy was 4 days (range: 0-29 days).

## Conclusions

We conclude that HZ a prevalent disease in Spain and that its frequency increases with age. Our findings are consistent with those from other European countries. The forthcoming introduction of a vaccine to prevent HZ warrants further large observational studies and pharmacoeconomical analyses.

## Abbreviations

HZ: Herpes zoster; VZV: Varicella zoster virus; PHN: Postherpetic neuralgia; BPI: Brief Pain Inventory; SD: Standard deviation; IQR: Interquartile range; CSISP: Centro Superior de Investigación en Salud Pública; CI: Confidence interval; HIV: Human immunodeficiency virus; GP: General practitioner.

## Competing interests

Unrestricted grants from Conselleria de Sanitat of the Generalitat Valenciana and Sanofi Pasteur MSD were used to support this research, but this financial support did not influence the analysis or interpretation of the data. JDD has received research funding from Sanofi Pasteur MSD and GlaxoSmithKline for attending advisory board meetings. MSR has been a consultant to Sanofi Pasteur MSD. The following authors declare that they have no competing interests: ACC, JPB and JNP.

## Authors' contributions

ACC and JDD contributed to patient recruitment, data collection, data analysis, and manuscript preparation. JPB and JNP contributed to the critical revision of the manuscript. MSR contributed to data analysis and manuscript preparation. All authors read and approved the final manuscript.

## Pre-publication history

The pre-publication history for this paper can be accessed here:

http://www.biomedcentral.com/1471-2296/11/33/prepub
